# Quantitative pupillometry as a sensitive biomarker for detecting subtle neurological impairment in mild traumatic brain injury – a pilot study

**DOI:** 10.1007/s10072-025-08405-2

**Published:** 2025-08-13

**Authors:** Dorothea Mitschang, Viktoriya Sydorenko, Daniel Kühlwein, Christopher Beynon, Alexander Younsi, Sandro M. Krieg, Pavlina Lenga

**Affiliations:** 1 Department of Neurosurgery, Heidelberg University Hospital, Im Neuenheimer Feld 400, 69120 Heidelberg, Germany; 2 Medical Faculty of Heidelberg University, Heidelberg, Germany; 3 Department of Neurosurgery, Mannheim University Hospital, Mannheim, Germany

**Keywords:** Traumatic brain injury, Quantitative pupillometry, Point of care evaluation, Prediction of neurological impairment

## Abstract

**Purpose:**

Mild traumatic brain injury accounts for roughly 55 million trauma cases globally each year. However, diagnosis and management remain challenging as conventional methods like cranial CT and the Glasgow Coma Scale do not reliably detect subtle neurological impairments. Quantitative Pupillometry has shown promise in severe traumatic brain injury, but data on its use in mild traumatic brain injury is limited. This study aims to evaluate whether Quantitative Pupillometry can detect subtle neurological dysfunction in patients with mild traumatic brain injury.

**Methods:**

We retrospectively analyzed 38 adult patients with mild traumatic brain injury admitted between December 2023 and October 2024. Quantitative Pupillometry was assessed in the emergency room using the NPi 200^®^ Pupillometer. Cranial CT was used to detect subarachnoid hemorrhage. Pupillary parameters (Neurological Pupil Index, constriction/dilation velocities, latency) and clinical data (GCS) were analyzed.

**Results:**

Mean age in our cohort was 61.8 years (SD 21.1). subarachnoid hemorrhage was present in 58% of patients (*n* = 22/38), with bleeding equally distributed between the hemispheres. In patients with subarachnoid hemorrhage, average pupillary dilation velocity was significantly lower compared to those without subarachnoid hemorrhage (0.7 mm/s vs. 1.1 mm/s, *p* = 0.043). Strong positive and significant correlations were found between pupillometric velocity parameters and GCS scores: bilateral constriction velocity (Sr = 0.9, *p* < 0.001) and dilation velocity (Sr = 0.8, *p* = 0.006). Multivariate regression analysis explained 73.8% of GCS variance, identifying increased pupillary latency and subarachnoid hemorrhage as significant predictors for worse GCS outcomes. ROC curve analysis confirmed the predictive value of subarachnoid hemorrhage presence (AUC = 0.8) and pupillary latency (AUC = 0.7).

**Conclusion:**

Quantitative Pupillometry, especially pupillary reflex velocities, is a sensitive tool for detecting subtle neurological impairments in mild traumatic brain injury patients who may appear clinically normal. This pilot study supports Quantitative Pupillometry as an effective tool for early identification of neurological deterioration, potentially improving triage and patient safety. These findings lay the foundation for further validation of Quantitative Pupillometry in mild traumatic brain injury assessment.

## Introduction

Mild traumatic brain injury (mTBI) accounts for roughly 80% of the 64–74 million new TBI cases occurring worldwide each year [1]. Despite being labeled “mild,” these injuries carry substantial burden and uncertainty: up to 15% of patients develop persistent post-concussive symptoms and functional impairment [1–4]. Early evaluation of mTBI is challenging, as diagnosis often relies on subjective symptoms and standard neurological examinations ​ [3–7]. Indeed, high-risk groups (e.g. athletes, military personnel) frequently depend on self-reported indicators of concussion, an approach with poor sensitivity for subtle brain dysfunction [7–12]. Conventional tools like the GCS and head CT have limitations in this context – a GCS of 13–15 and normal CT do not preclude significant neural injury or future deterioration [11, 12]. In practice, occult deficits in cognition or autonomic function may go undetected in mTBI, underscoring the need for more sensitive, objective prognostic measures ​ [12].

QP has emerged as an attractive adjunct for objective neurologic assessment. Unlike the traditional flashlight pupillary exam – which is subjective and notoriously unreliable [13–15] – QP uses automated infrared devices to precisely measure pupillary light reflex dynamics. Handheld pupillometers generate reproducible metrics such as pupillary size, constriction latency, velocity of constriction and dilation, and the Neurological Pupil index (NPi)​ [14, 16]. In intensive care and severe TBI settings, these quantitative pupillary metrics have been validated as early indicators of neurologic worsening. For example, an abnormally low NPi (<3) or a slowing of pupillary response correlates with episodes of intracranial hypertension and worse outcomes in severe TBI [17]. Similarly, recent studies show that diminished pupillary reactivity is linked with greater injury severity – a reduced dilation velocity is associated with lower GCS scores and the need for urgent intervention after TBI [10, 18, 19]. These findings highlight the prognostic potential of pupillometry in acute brain injury management [10, 18–22]. It remains unclear, however, whether quantitative pupillometry can meaningfully stratify risk in patients with mild TBI. Most evidence to date comes from severe TBI or controlled concussion research rather than the emergency or critical care evaluation of mTBI. Early data from sports concussion suggest that even mild brain injury can alter pupillary reflexes – concussed adolescents demonstrate larger baseline pupil diameters and faster constriction/dilation velocities compared to uninjured peers [18, 19]. Such observations make QP a promising objective biomarker in concussion and mTBI [10, 18–22]. Yet in clinical practice, the utility of pupillometry for mTBI remains poorly defined, with no consensus on its ability to detect subtle neurologic deterioration or predict outcome in this “mild” spectrum of TBI.

In this study, we investigate the prognostic value of QP in mTBI. We evaluate whether pupillary indices – including NPi, constriction and dilation velocities, and latency – can predict impaired clinical status in mTBI patients. Our aim is to determine if objective pupillary metrics can augment current assessment tools, thereby improving early identification of patients at risk for neurologic impairment despite an ostensibly mTBI.

## Methods

### Study design and data collection

We retrospectively collected data of patients presenting with mTBI in our ER between December 2023 and October 2024. The study was approved by the local ethics committee (registration number: S-788/2021) and conducted in accordance with the Declaration of Helsinki. As previously defined by the World Health Organization, head trauma is considered a mTBI if post traumatic GCS is between13-15 and one or more of the following symptoms is present: (1) post-traumatic amnesia, (2) loss of consciousness < 30 min, (3) impaired mental state at time of accident (confusion, disorientation, etc.), and/or (4) transient neurological deficit [16, 23]. In case of radiographic proof of SAH in cranial CT scan, patients were admitted for clinical observation. Only Patients aged ≥18 years were enrolled. The exclusion criteria were as follows: age < 18 years, GCS < 13. Clinical and Imaging Data were accessed via our institutions database.

### Pupillometric data

Pupillometry was conducted using the NPi 200^®^ Pupillometer (Neuroptics, Laguna Hill, USA), a handheld infrared device that automatically records and analyzes pupil dynamics over a 3-s period. The NPi is an algorithm-generated score ranging from 0 to 5, where values between 4 and 5 are considered within normal limits, and values below 4 indicate an abnormal pupillary light reflex, potentially reflecting neurological dysfunction [13, 24]. The NPi evaluates variables such as pupil size, latency, constriction velocity, and dilation velocity to provide an objective assessment of pupillary function.

Pupillometry was performed in the ER for all patients with traumatic brain injury. Since automated pupillometry is associated with a low interobserver variability, we did not perform repeated examinations at any given time point [24].

### Statistical analysis

Categorical variables are presented as numbers and percentages. Continuous variables are presented as mean and standard deviation (SD). Patient characteristics and pupillometric data were compared using independent t-tests for continuous variables and fishers exact test for categorical variables. In the second-stage analysis correlation between pupillometric data as mentioned above and GCS was calculated by Spearman’s correlation. Spearman Rho correlations coefficients were interpreted as follows: no correlation 0 to < 0.1, weak correlation 0.1 to < 0.3, moderate correlation 0.3 to < 0.5, and strong correlation ≥ 0.5. Finally, a multivariate linear regression analysis was performed with GCS as dependent variable and pupillometric parameters as independent variables. Statistical significance was set at a p-value ≤ 0.05. Statistical analysis was performed using SPSS software (version 24.0.0.0, IBM Corp., Armonk, NY, USA).

## Results

### Demographic characteristics and neurological status

In total, 38 adult patients (≥ 18 years) with mTBI, defined by GCS scores ranging from 13 to 15, were enrolled between December 2023 and October 2024 (Table 1). SAH was identified via cranial CT scan in 22 patients (58%). The overall mean age was 61.8 years (SD 21.1). GCS score was 15 (IQR 1.0) across the entire cohort, including patients without SAH. Patients diagnosed with SAH had a slightly lower median GCS of 14 (IQR 4.0), though this difference was not statistically significant. Among patients presenting with SAH, bleeding was evenly distributed between the right and left cerebral hemispheres, each accounting for exactly half (*n* = 11 per hemisphere) (Table 2).

**Table 1:** Demographics and pupillometric parameters of patients with mild traumatic brain injury

Parameter	Overall (*n* = 38)	SAH (*n* = 22)	No SAH (*n* = 16)	*p*-value
Mean Age (SD)	61.8 (21.1)	66.2 (17.4)	55.9 (24)	0.672
Sex, (%)				0.511
Male	24 (63)	15 (68)	9 (56)	
Female	14 (37)	7 (32)	7 (44)	
Median GCS (IQR)	15 (1)	14 (4.0)	15 (0.0)	0.487
Side of SAH, (%)				
Right	-	11 (50.0)	-	
Left	-	11 (50.0)	-	
Pupillometry Findings				
Bilateral				
Mean NPi (SD)	4.3 (0.6)	4.3 (0.7)	4.4 (0.3)	0.438
Mean Size (before constriction), mm (SD)	3.4 (1.0)	3.1 (0.8)	3.9 (1.1)	0.264
Mean Min Pupil Size, mm (SD)	2.4 (0.5)	2.3 (0.5)	2.6 (0.6)	0.478
Mean Constriction Index, Percentage (SD)	27.4 (9.5)	23.7 (10.3)	32.1 (5.5)	0.184
Mean Constriction Velocity, mm/s (SD)	1.9 (0.9)	1.5 (0.8)	2.4 (0.7)	0.162
Mean Max Constriction Velocity, mm/s (SD)	2.9 (1.4)	2.3 (1.1)	3.7 (1.3)	0.141
Mean Dilatation Velocity, mm/s (SD)	0.9 (0.4)	0.7 (0.3)	1.1 (0.3)	**0.043**
Mean Latency, s (SD)	0.3 (0.0)	0.3 (0.0)	0.2 (0.0)	0.181
Right				
Mean NPi (SD)	4.3 (0.8)	4.2 (1.0)	4.4 (0.3)	0.254
Mean Size (before constriction), mm (SD)	3.4 (1.2)	3.1 (1.0)	3.9 (1.1)	0.405
Mean Min Pupil Size, mm (SD)	2.4 (0.6)	2.3 (0.6)	2.6 (0.7)	0.595
Mean Constriction Index, Percentage (SD)	26.8 (10.3)	22.7 (10.9)	32.2 (6.2)	0.248
Mean Constriction Velocity, mm/s (SD)	1.9 (0.9)	1.5 (0.9)	2.4 (0.8)	0.184
Mean Max Constriction Velocity, mm/s (SD)	3.0 (1.5)	2.4 (1.4)	3.7 (1.3)	0.142
Mean Dilatation Velocity, mm/s (SD)	0.9 (0.4)	0.8 (0.3)	1.1 (0.3)	**0.044**
Mean Latency, s (SD)	0.3 (0.1)	0.3 (0)	0.2 (0.1)	0.236
Left				
Mean NPi (SD)	4.4 (0.5)	4.4 (0.6)	4.4 (0.3)	0.986
Mean Size (before constriction), mm (SD)	3.4 (1.0)	3.1 (0.8)	4.0 (1.1)	0.426
Mean Min Pupil Size, mm (SD)	2.4 (0.6)	2.3 (0.5)	2.6 (0.6)	0.750
Mean Constriction Index, Percentage (SD)	27.9 (9.8)	24.8 (10.7)	32.1 (5.5)	0.330
Mean Constriction Velocity, mm/s (SD)	1.9 (0.9)	1.4 (0.8)	2.4 (0.8)	0.209
Mean Max Constriction Velocity, mm/s (SD)	2.9 (1.4)	2.3 (1.1)	3.7 (1.3)	0.162
Mean Dilatation Velocity, mm/s (SD)	0.9 (0.4)	0.7 (0.3)	1.1 (0.3)	**0.023**
Mean Latency, s (SD)	0.3 (0.0)	0.3 (0)	0.2 (0.0)	0.567

*GCS *Glasgow Coma Scale, *IQR* Interquartile Range, *NPi* Neurological Pupil Index, *SAH* Subarachnoid Hemorrhage, *SD* Standard Deviation

**Table 2:** Correlations analysis between GCS of patients with SAH and pupillometry findings

Parameter	Spearman-Rho	*p*-value
NPi		
Bilateral	−0.1	0.363
Right	−0.1	0.474
Left	−0.4	0.127
Pupil Size (before constriction)		
Bilateral	0.8	**0.007**
Right	0.7	**0.022**
Left	0.9	**0.001**
Minimal Pupil Size		
Bilateral	0.7	**0.014**
Right	0.7	**0.045**
Left	0.5	**0.042**
Constriction Index		
Bilateral	0.7	**0.028**
Right	0.8	**0.006**
Left	0.5	0.106
Average Constriction Velocity		
Bilateral	0.9	**< 0.001**
Right	0.9	**0.001**
Left	0.7	**0.010**
Minimal Constriction Velocity		
Bilateral	0.9	**0.001**
Right	0.9	**< 0.001**
Left	0.7	**0.010**
Average Dilatation Velocity		
Bilateral	0.8	**0.006**
Right	0.9	**< 0.001**
Left	0.7	**0.010**
Latency		
Bilateral	−0.2	0.333
Right	−0.1	0.435
Left	−0.2	0.279

*GCS *Glasgow Coma Scale, *NPi* Neurological Pupil Index, *SAH* Subarachnoid Hemorrhage

### QP Parameters

Significant differences emerged in pupillometric parameters between patients with and without SAH. Bilateral average pupillary dilatation velocity was significantly lower in patients with SAH (mean = 0.7 mm/s, SD 0.3) compared to patients without SAH (mean = 1.1 mm/s, SD 0.3; *p* = 0.043). This reduction was more pronounced in the left pupil (mean = 0.7 mm/s, SD 0.3 vs. 1.1 mm/s, SD 0.3; *p* = 0.023) compared to the right pupil (mean = 0.8 mm/s, SD 0.3 vs. 1.1 mm/s, SD 0.3; *p* = 0.044). Other pupillometric parameters did not significantly differ between groups.

### Correlation between pupillometric parameters and GCS

Correlation analyses revealed several significant associations between pupillometric parameters and GCS scores. Higher GCS scores were significantly associated with larger pupil sizes in bilateral measurements (S_r_ = + 0.8, *p* = 0.007), and individually in the right (S_r_ = + 0.7, *p* = 0.022) and left pupils (S_r_ = + 0.7, *p* = 0.014). Minimal pupil diameters also positively correlated with higher GCS scores, bilaterally (S_r_ = + 0.7, *p* = 0.014), and individually in the right (S_r_ = + 0.6, *p* = 0.045) and left eyes (S_r_ = + 0.5, *p* = 0.042). The pupillary constriction index showed positive correlations with GCS bilaterally (S_r_ = 0.7, *p* = 0.028) and strongly in the right eye (S_r_ = 0.8, *p* = 0.006), though not significantly in the left eye (S_r_ = 0.5, *p* = 0.106). Velocity parameters demonstrated the strongest correlations with GCS: average constriction velocity correlated positively bilaterally (S_r_ = 0.9, *p* < 0.001), and individually in the right (S_r_ = 0.9, *p* = 0.001) and left eyes (S_r_ = 0.7, *p* = 0.010). Minimal constriction velocity similarly showed robust positive correlations (bilateral S_r_ = 0.9, *p* = 0.001; right S_r_ = 0.9, *p* < 0.001; left S_r_ = 0.7, *p* = 0.010). Average dilation velocity correlated positively with GCS bilaterally (S_r_ = 0.8, *p* = 0.006), in the right (S_r_ = 0.9, *p* < 0.001), and left eyes (S_r_ = 0.7, *p* = 0.010). There were no significant correlations between GCS and Neurological Pupil Index (NPi) scores or pupillary latency measures.

### Multivariate linear regression analysis

Multivariate linear regression analysis explained approximately 73.8% (R² = 0.738) of the variance in GCS scores, demonstrating a strong predictive value of selected pupillometric and clinical variables (Table 3). Pupillary latency in the left eye was associated with worse GCS outcomes (coefficient = −18.5, *p* = 0,688), indicating prolonged latency predicts lower GCS scores. Conversely, larger minimum pupil diameter bilateral positively predicted better GCS scores (coefficient = + 4.8; *p* = 0.042), however these findings did not reach statistical significance. Bilateral pupillary constriction index showed a positive correlation with higher GCS scores (coefficient = 0.058, *p* = 0.028). The presence of SAH was significantly associated with lower GCS scores (Coefficient = −0.697, *p* = 0.027). Other variables, such as NPi scores, and pupil sizes, also contributed modestly yet meaningfully to the model.

**Table 3:** Multivariate regression analysis of pupillometric and clinical predictors of Glasgow coma scale scores in mild traumatic brain injury

Parameter	Coefficient	Absolute Coefficient	*p*-value
NPi			
Bilateral	−0.1	0.1	0.527
Right	−3.7	3.7	0.328
Left	3.6	3.6	0.528
Pupil Size (before constriction)			
Bilateral	−2.8	2.8	0.079
Right	0.6	0.6	0.067
Left	−6.2	6.2	0.088
Minimal Pupil Size			
Bilateral	4.8	4.8	0.572
Right	−6.9	6.9	0.674
Left	16.5	16.5	0.455
Constriction Index			
Bilateral	0.3	0.3	**0.028**
Right	0.1	0.1	0.056
Left	0.3	0.3	0.045
Average Constriction Velocity			
Bilateral	−0.2	0.2	0.534
Right	0.1	0.1	0.877
Left	−0.3	0.3	0.677
Minimal Constriction Velocity			
Bilateral	−0.5	0.5	0.372
Right	0.5	0.5	0.370
Left	−1.5	1.5	0.418
Average Dilatation Velocity			
Bilateral	−9.2	9.2	0.657
Right	8.0	8.0	0.475
Left	5.3	5.3	0.238
Latency			
Bilateral	−5.0	5.0	0.655
Right	8.5	8.5	0.724
Left	−18.5	18.5	0.688
SAH	−1.2	1.2	**0.027**
Headache and Nausea	0.8	0.8	0.788

*GCS* Glasgow Coma Scale, *NPi* Neurological Pupil Index, *SAH* Subarachnoid Hemorrhage

### ROC curve analysis

**Fig. 1:**
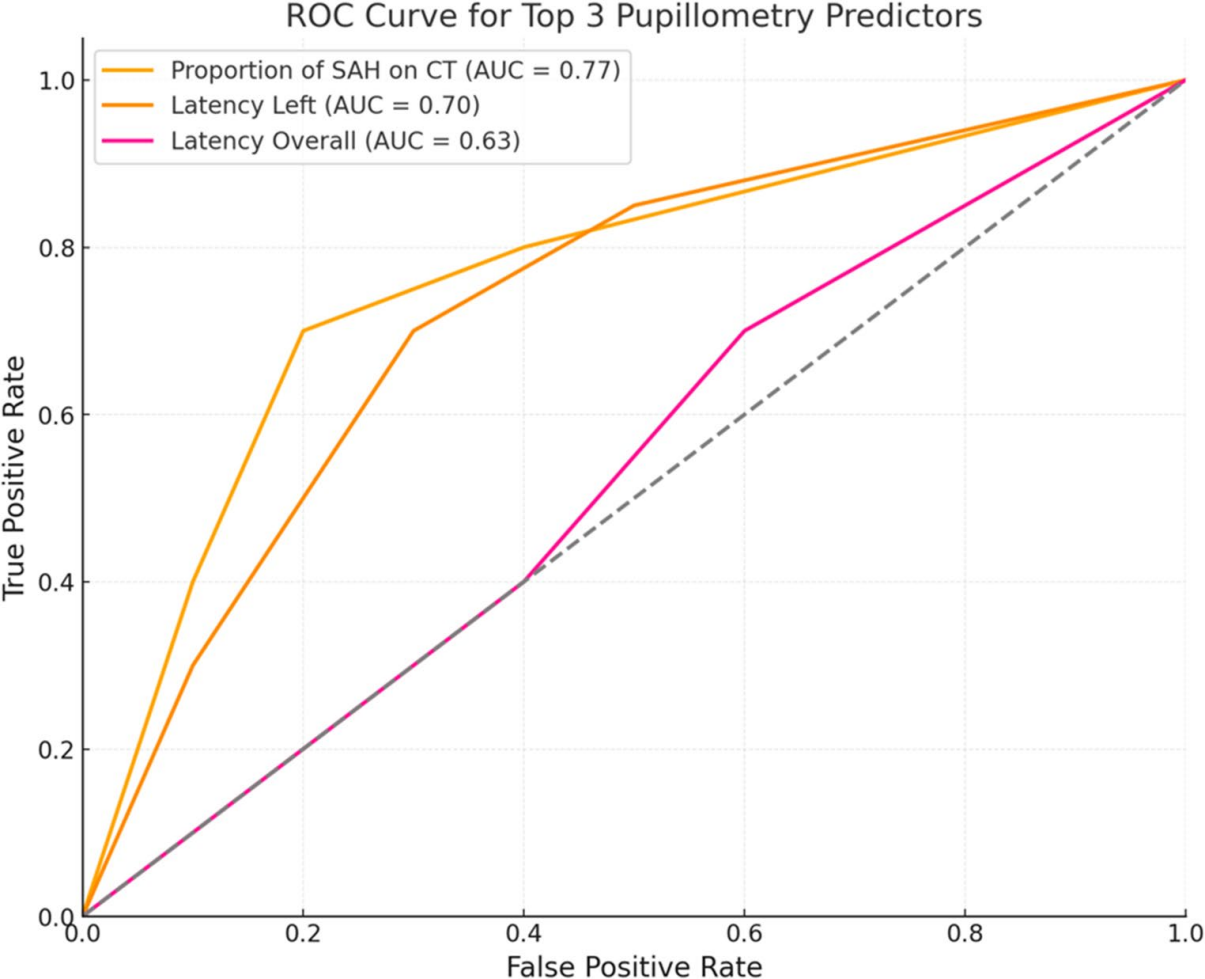
ROC Curve Analysis of predictive value of SAH, Latency in the Left Pupil and Bilateral Latency. Abbreviations:AUC = Area under the Curve, ROC = Receiver Operating Characteristic, SAH = Subarachnoid Hemorrhage,

ROC curve analysis confirmed the predictive significance of these parameters, identifying SAH presence (AUC = 0.8), left pupillary latency (AUC = 0.7), and bilateral pupillary latency (AUC = 0.6) as the strongest predictors of GCS outcomes in patients with mTBI (Fig. 1).

## Discussion

In our cohort of mTBI patients, QP effectively differentiated subtle neurological impairments that conventional neurological assessments might overlook. Importantly, pupillary reflex velocities—particularly pupillary constriction and dilation speeds—emerged as the most sensitive indicators of impaired neurologic status. We observed significantly slower pupillary velocities even among patients with slightly reduced consciousness (GCS 13–14) compared to fully alert individuals. Conversely, the NPi, a composite metric of overall pupil reactivity, remained within normal limits for most patients in our mild-injury cohort. These findings suggest that dynamic pupillary parameters (such as how quickly the pupil reacts to light stimuli) are more sensitive indicators of mild diffuse brain injury than static pupil size or binary measures of pupillary reactivity. Thus, subtle slowing in pupillary response velocities may serve as an early and objective biomarker of neural dysfunction in patients whose clinical presentation (near-normal GCS) might otherwise suggest negligible injury.

### Comparison with previous atudies

Our findings align with emerging literature highlighting that QP metrics correlate strongly with TBI severity. In a large cohort of over 2,000 brain injury cases, Thakur et al. reported progressively slower pupillary dilation velocities as injury severity increased, with higher GCS scores consistently associated with faster dilation speeds. Specifically, patients with mild injuries (GCS 13–15) had significantly faster pupil dilation (~ 0.85–0.87 mm/s) compared to moderate (~ 0.72 mm/s) or severe (~ 0.50 mm/s) cases [25]. Similarly, Traylor et al. identified slower pupil dilation velocities in acute TBI patients who experienced loss of consciousness or required urgent intervention, but, notably, found no significant acute-phase correlation between NPi scores and neurologic status [15]. This supports our observation that while the composite NPi often remains normal in mild cases, subtle velocity alterations can still indicate neuronal dysfunction. The prognostic value of NPi compared to pupillary velocity metrics has indeed varied across the severity spectrum. In severe TBI patients, Jahns et al. observed significant reductions in NPi (< 3) linked to sustained intracranial hypertension and poorer long-term outcomes [26]. Additionally, a multicenter analysis demonstrated enhanced outcome prediction when pupillary reactivity (NPi or manual assessment) was incorporated into the GCS, underscoring NPi’s prognostic importance when severe impairment is present [27]. However, in mild TBI cases—such as our study cohort—few patients cross the abnormal NPi threshold, limiting its sensitivity. One study reported high specificity (92%) but only moderate sensitivity (~ 51%) of an abnormal NPi (< 3) in predicting short-term neurological deterioration [15]. This aligns with our findings and further emphasizes that normal NPi scores in mild TBI do not necessarily rule out subtle neurologic injury.

Our results are further corroborated by studies of concussion, which show pupillary changes even without overt neurological deficits. Research on adolescent athletes demonstrated significant acute reductions in pupillary constriction amplitude and slower pupillary velocities immediately post-concussion compared to preseason baselines [28]. These pupillary alterations were concussion-specific and absent in athletes who had not sustained head impacts [2, 28]. Notably, even subclinical head impacts—high-acceleration events without symptoms—produced measurable pupillary changes, suggesting that QP can detect neurologic effects otherwise hidden from clinical detection [11, 18, 22]. Moreover, evidence indicates pupillary changes can persist into the subacute recovery period. Master et al. reported enlarged baseline pupils and increased constriction amplitude in adolescents one month post-concussion [18], possibly reflecting autonomic rebalancing during recovery. Military studies similarly demonstrated prolonged constriction latency and slower velocities weeks after mild injuries, underscoring that pupillary responses evolve over time following mTBI [11, 18].

Finally, emerging data using advanced technologies reinforce these findings. A recent cohort study employing smartphone-based pupillometry differentiated acute mTBI patients (GCS 15, normal head CT) from healthy controls with high accuracy (~ 93%), driven primarily by reduced pupillary change and significantly slower mean constriction velocities (nearly 40% slower than controls) [29]. The success of these portable, widely accessible pupillometry approaches lends confidence to the robustness and reproducibility of quantitative pupillary deficits we observed, suggesting genuine neurobiological disruption associated with mild TBI rather than isolated findings.

### Pathophysiological considerations

The observed slowing in pupillary reflex velocities likely reflects transient impairment in the complex neural pathways mediating the pupillary light reflex. Under physiological conditions, pupil constriction and dilation involve an integrated response orchestrated primarily through cranial nerves II (optic) and III (oculomotor), along with critical modulation from brainstem nuclei responsible for arousal and autonomic control. mTBI, although clinically subtle, can produce diffuse axonal stretching or microscopic injury affecting white matter connectivity, particularly within the midbrain and brainstem regions involved in pupillary reflex circuits. Such diffuse microstructural changes could result in the slower conduction velocity observed clinically as delayed pupillary constriction or dilation. Furthermore, decreased brainstem arousal associated with mTBI—potentially due to transient metabolic disruption or neurotransmitter imbalance—could further exacerbate slowed pupillary kinetics, as the pupillary reflex is sensitive to changes in central arousal states. Given that the NPi primarily detects pronounced abnormalities typically resulting from structural or severe functional injury, its relative insensitivity to subtle or diffuse injuries aligns with current understanding of its clinical utility [30]. Instead, the dynamic pupillary parameters captured by QP (e.g., latency and velocity) appear more responsive to mild disruptions within neural circuits. Therefore, even minor perturbations in brainstem and cranial nerve pathways resulting from mild neurotrauma are sufficient to induce measurable changes in autonomic reflex control. This highlights the neurophysiological basis supporting pupillary reflex velocities as sensitive biomarkers capable of detecting mild yet clinically meaningful neural dysfunction after mTBI.

### Clinical relevance

QP significantly enhances the clinical evaluation of TBI by providing objective, reproducible measurements of pupil size and reaction. It reduces the subjectivity and inter-observer variability inherent in manual pupil assessments, where error rates can exceed 20% for detecting anisocoria and even 50% for accurately judging pupil reactivity [15, 19]. This advantage is particularly relevant in mTBI and concussion, where standard clinical assessments such as GCS or symptom checklists often appear normal or may be influenced by patient underreporting [7, 11, 28]. By quantitatively detecting subtle neurologic impairments—such as slightly slowed pupillary constriction—even in patients denying symptoms or in those with confounding factors like sedation or intoxication, QP provides clinicians with a physiologic biomarker of brain injury. In emergency and acute care settings, pupillometry facilitates early identification of patients at risk of clinical deterioration. Previous research has shown that abnormal pupillary readings, particularly NPi values below 3, reliably predict the need for neurosurgical intervention (e.g., ICP monitoring, craniectomy) [5]. Although our cohort consisted mainly of mild TBI patients who mostly showed normal NPi values, even subtle abnormalities or unexpected slowing in pupillary reflexes could indicate occult intracranial pathology requiring closer observation or escalation of care. Conversely, in ambiguous clinical situations (e.g., borderline GCS or subtle CT abnormalities), a normal NPi might reassure clinicians that conservative management is sufficient [10]. Thus, integrating pupillometry into early trauma assessments can improve triage decisions, ensuring that patients who truly require intensive monitoring or intervention receive prompt attention, while others avoid unnecessary resource-intensive care. Beyond the initial evaluation, QP plays an increasingly important role in ongoing neuromonitoring within neurocritical care units. Serial pupillary measurements, tracking NPi and velocity trends over time, enable the early detection of secondary neurologic insults such as rising intracranial pressure or impending herniation. A progressive slowing of pupillary velocity or declining NPi often precedes clinical deterioration, providing a crucial window for early therapeutic intervention [25]. Although mTBI patients typically do not require intensive care, occasional cases of delayed neurological worsening (e.g., delayed hematoma) underscore the value of pupillometric surveillance, enabling clinicians to detect subtle deterioration before routine neurological checks or imaging studies indicate a problem [25, 31]. This proactive approach to neuromonitoring can guide decisions about treatment escalation or de-escalation, such as the administration of hyperosmolar therapy or reducing sedation levels to allow more thorough neurological evaluation. From a practical standpoint, our findings and supporting literature suggest clear clinical benefits of incorporating pupillometry into mTBI management. In concussion clinics, emergency departments, or sports medicine settings, objectively slowed pupillary reflexes in patients who appear clinically mild can prompt closer follow-up, extended observation, or early referral for specialized neurological evaluation. Pupillometric data may also inform return-to-play or discharge decisions, identifying patients who remain at risk despite subjective improvement. Overall, pupillometry adds a valuable objective tool to traditional clinical evaluation—enhancing the identification, monitoring, and management of subtle neurologic dysfunction following mTBI.

## Limitations

Several limitations should be considered when interpreting these findings. Our study involved a modest, single-center sample, potentially limiting the generalizability of results. Additionally, the use of the GCS as our primary outcome measure has known ceiling effects in mTBI, potentially underestimating subtle neurological or cognitive deficits. Future studies could benefit from assessing longer-term functional or neuropsychological outcomes beyond the acute GCS. The retrospective observational design introduced potential biases, including variability in timing of pupillary assessments and possible influence of unmeasured confounders (e.g., intoxication, sedation, or ocular injuries). Individual baseline variability in pupil responses was also not accounted for, and device-specific differences (single brand pupillometer) could limit comparability with other pupillometry studies. Finally, variability across existing studies in pupillary metrics, definitions of abnormal thresholds, and severity ranges highlights the need for standardized protocols and prospective validation. Thus, our findings should be interpreted as preliminary evidence supporting pupillometry in mTBI, requiring confirmation and refinement through larger multicenter studies.

## Future directions

Future research should validate these findings in larger, multicenter cohorts, establishing normative ranges and confirming predictive pupillometry metrics in mTBI. Longitudinal studies correlating early pupillary changes with outcomes such as neurocognitive function, persistent symptoms, or return-to-work would strengthen the clinical case for routine pupillometry use. Further development of prognostic models combining pupillary metrics (e.g., NPi, dilation velocity) with traditional clinical data (age, GCS, CT findings) is also promising. Prospective validation could enhance risk stratification and identify patients needing closer monitoring or specific interventions. Portable, smartphone-based pupillometers provide a practical route toward wider adoption, including emergency, sports, and military settings. Studies evaluating these devices in real-world scenarios could demonstrate their practical clinical utility. Finally, randomized trials evaluating pupillometry-guided management decisions versus standard care, alongside mechanistic studies linking pupillary changes to biomarkers and imaging, would clarify pupillometry’s direct clinical impact and biological relevance, solidifying its integration into routine mTBI management.

In detail, this pilot study provides preliminary findings and is the foundation for future evaluation und validation of QP as an essential component of TBI evaluation in a larger cohort and multicentric approach. We plan to extend our data by including QP as a standard diagnostic tool within triage evaluation for all patients presenting in our ER with mTBI and thus expanding our future cohort.

## Conclusion

This pilot study demonstrates that QP, specifically pupillary reflex velocities, effectively detects subtle neurological impairments in mTBI patients who may otherwise appear clinically normal on routine assessments. Pupillary velocities emerged as sensitive, objective indicators, revealing significantly slower pupil responses even among patients with minimally depressed consciousness (GCS 13–14). These findings underscore the value of pupillometry as a reliable biomarker of neural dysfunction in patients traditionally classified as “mild”, potentially improving early identification, triage decisions, and patient safety. Given its non-invasive nature, ease of use, and strong predictive capability, QP has the potential to become an essential component of initial trauma evaluations, concussion management protocols, and ongoing clinical monitoring strategies.

## Data Availability

The datasets generated during and/or analyzed during the current study are available from the corresponding author on reasonable request.
